# Systolic and diastolic function during cycling at the respiratory threshold between elderly and young healthy individuals

**DOI:** 10.1038/s41598-022-07933-7

**Published:** 2022-03-09

**Authors:** Sara Magnani, Gabriele Mulliri, Silvana Roberto, Giovanna Ghiani, Fabio Sechi, Silvia Stagi, Elisabetta Marini, Pier Paolo Bassareo, Marty D. Spranger, Antonio Crisafulli

**Affiliations:** 1 Department of Medical Sciences and Public Health, SportsPhysiologyLaboratory, University of Cagliari, Via Porcell 4, 09124 Cagliari, Italy; 2 International PhD in Innovation Sciences and Technologies, University of Cagliari, Cagliari, Italy; 3 Department of Life and Environmental Sciences, University of Cagliari, Cagliari, Italy; 4 School of Medicine, Mater Misericordiae University Hospital, University College of Dublin, Dublin, Ireland; 5 Department of Physiology, Michigan State University, East Lansing, USA

**Keywords:** Ageing, Circulation, Physiology, Cardiology

## Abstract

The hemodynamic consequences of aging have been extensively investigated during maximal incremental exercise. However, less is known about the effects of aging on hemodynamics during submaximal steady-state exercise. The aim of the present investigation was to compare the hemodynamics of healthy elderly and young subjects during an exercise bout conducted at the gas threshold (GET) intensity. Two groups of healthy, physically active subjects were studied: the elderly group—EG (n = 11; > 60 years old) and the young group—YG (n = 13; < 35 years old). Both groups performed a 5-min rectangular exercise test at the GET intensity. Hemodynamics were measured using echocardiography. The main finding was that stroke volume responses were higher in the YG than the EG (72.5 ± 16.7 vs. 52.4 ± 8.4 ml, respectively). The increased stroke volume capacity in the YG was the consequence of a greater capacity to increase cardiac preload and contractility and, to a lesser extent, to reduce systemic vascular resistance. Importantly, the atrial contribution to ventricular diastolic filling was substantially higher in the YG when compared to the EG.

## Introduction

Among the various physiological factors influencing exercise capacity, cardiac pumping is of paramount importance^1^. The cardiovascular response during exercise encompasses complex changes in heart rate (HR), myocardial contractility, preload, and afterload^2–7^. This response is influenced by many factors, including aging, which substantially reduces the capacity to augment cardiac output (CO), and therefore to exercise^1,8–11^. Several concurring phenomena are responsible for the age-related reduction in the pumping capacity of the heart. It is well established that maximal HR progressively decreases with age, consequently resulting in proportional reductions in maximal CO, maximal oxygen uptake (${\dot{\text{V}}}$O_2max_), and maximal workload (W_max_)^12,13^. In contrast, whether stroke volume (SV) is preserved with aging remains controversial as the limited literature is conflicting^9,13–15^.

In some investigations conducted in healthy elderly individuals of both sexes, it was found that the decline in maximal CO during dynamic exercise was entirely due to the reduction in HR, since SV did not decline with age^14^. Importantly, however, aging did affect the processes by which the SV level was achieved in these studies. Specifically, older individuals showed a blunted capacity to reduce left ventricular end-systolic volume (ESV), and to increase ejection fraction (EF) in response to effort, however this deficit was offset by increasing end-diastolic volume (EDV) via the Frank-Starling mechanism. This increase in preload was made possible due to the slower HR, and subsequent longer diastolic interval, as compared to young subjects^14^. The underlying mechanisms for the age-associated reduction in maximal EF are multifactorial and likely include impaired myocardial performance, increased vascular resistance, impaired autonomic nervous system modulation of myocardial contractility, and an intrinsic impairment in cardiomyocyte contractile function^1,9,14^ Moreover, available data suggest that diastolic function also deteriorates with age, likely due to the age-associated increases in cardiac stiffness and impairment in ventricular relaxation^16–20^.

While hemodynamic differences between elderly and young subjects during incremental exercise tests up to exhaustion have been extensively studied, less is known about the hemodynamic differences during submaximal steady-state exercise. Some previous studies have examined the effect of aging during exercise echocardiography performed at submaximal workloads during cycling in the supine or in the reclimbing position at 60°. Workloads varied from 30 to 60% of maximal workload, or it was set to reach a fixed level of HR^21–24^. However, to the best of our knowledge, there are no studies comparing individuals of different ages when they exercise at the same subjective submaximal level of effort identified with standard methods. Specifically, we could not find any information about the effect of ageing during effort at constant workload at the intensity of the gas exchange threshold (GET), which represents a useful tool to discriminate the transition from moderate to heavy exercise. Importantly, steady-state in cardio-pulmonary and metabolic variables cannot be reached with exercise performed above the intensity of GET, so exercise bouts above GET can not be sustained for long periods^25^. Moreover, while ${\dot{\text{V}}}$O_2max_ declines with age, paralleling that of HR and CO, the reduction in GET is delayed, suggesting that the cardiovascular response during submaximal exercise is well-preserved with aging^25,26^. However, the effects of aging on hemodynamics during submaximal steady-state exercise at the intensity of the GET have yet to be elucidated. Reduction in ventricular relaxation and increase in the atrial component of ventricular diastolic filling have been repeatedly reported with senescence at rest as well as during exercise-induced tachycardia^13,14,16,17^. Diastolic dysfunction is actually a part of the aging process of the heart, which leads to an impairment in ventricular early filling with a concomitant shift towards atrial filling as a mechanism through which SV in preserved^23^. However, to what extend these cardiovascular changes affect the cardiovascular response during submaximal exercise is largely unknown. It should be considered that the capacity to maintain and/or augment SV is pivotal for as effective cardiovascular response to exercise and to guarantee an optimal muscle perfusion, especially in older individuals where the chronotropic response is reduced. This has been demonstrated for effort up to maximum, but it is possible to hypothesize that an impaired diastolic filling negatively affects hemodynamic already at submaximal exercise.

Therefore, we devised the present investigation to compare hemodynamics of two groups of healthy individuals, the elderly (EG) and the young group (YG), during an exercise bout conducted at the same relative submaximal workload, i.e., at the GET intensity. Specifically, we hypothesized that elderly individuals would have a reduced capacity to increase SV during exercise at the GET intensity due to their impaired diastolic and systolic function as compared to young individuals. Moreover, reduced capacity to increase ventricular filling and emptying rates limit increases in CO and may, at least in part, explain a lower workload at GET.

## Results

The study protocol was completed by all the subjects. Table 1 shows the anthropometric data and the results of the preliminary cardiopulmonary test (CPT). Subjects in the YG were taller than those in the EG, but there was no difference between groups in body mass and body mass index (BMI). Maximum HR (HR_max_), W_max_, ${\dot{\text{V}}}$O_2max_ (expressed in terms of absolute values as well as indexed by body mass), maximum carbon dioxide production (${\dot{\text{V}}}$CO_2max_), and maximum pulmonary ventilation (V_Emax_) were all higher in the YG compared to the EG, whereas the maximum respiratory exchange ratio (RER) was not significantly different. HR, workload, ${\dot{\text{V}}}$O_2_, and ${\dot{\text{V}}}$CO_2_ at GET were significantly higher in the YG than in the EG, while RER and V_E_ were similar. GET occurred at 51.76 ± 8.76 and 73.91 ± 13.18% of W_max_ in the YG and EG, respectively (p < 0.0001).

**Table 1:** Anthropometric characteristics of groups together with results of the cardiopulmonary test. *EG* elderly group (n = 11, 7 females), *YG* young group (n = 13, 7 females), *GET* gas exchange threshold. Values are mean ± SD.

	EG	YG	*p* value
Age (years)	65.1 ± 4.7	29.8 ± 4.0	< 0.0001
Height (cm)	161.0 ± 8.6	169.3 ± 8.4	0.025
Body mass (kg)	61.3 ± 7.4	64.8 ± 9.7	0.348
Body mass index (kg m^2^)	23.7 ± 2.4	22.5 ± 2.4	0.254
Maximum heart rate (bpm)	145.8 ± 18.2	177.9 ± 9.2	< 0.0001
Maximum workload (W_max_)	115.4 ± 45.6	206.9 ± 51.2	< 0.0001
Maximum O_2_ uptake (ml min^−1^)	1419 ± 558	2191 + 648	0.005
Maximum O_2_ uptake/kg (ml min^−1^ kg^−1^)	22.6 ± 6.6	32.9 ± 5.6	< 0.001
Maximum CO_2_ production (ml min^−1^)	1794 ± 661	2894 ± 919	0.003
Maximum respiratory exchange ratio	1.27 ± 0.13	1.31 ± 0.09	0.384
Maximum pulmonary ventilation (l min^−1^)	54.4 ± 22.4	83.3 ± 27.5	0.011
Heart rate at GET (bpm)	126.6 ± 13.2	140.6 ± 9.2	0.003
Workload at GET (W)	81.8 ± 23.5	105.0 ± 23.9	0.026
O_2_ uptake at GET (ml min^−1^)	1089 ± 338	1368 ± 274	0.036
CO_2_ production at GET (ml min^−1^)	1093 ± 343	1375 ± 310	0.046
Respiratory exchange ratio at GET	1.01 ± 0.03	1.00 ± 0.07	0.700
Pulmonary ventilation at GET (l min^−1^)	33.6 ± 10.4	29.2 ± 8.6	0.271

**Figure 1:**
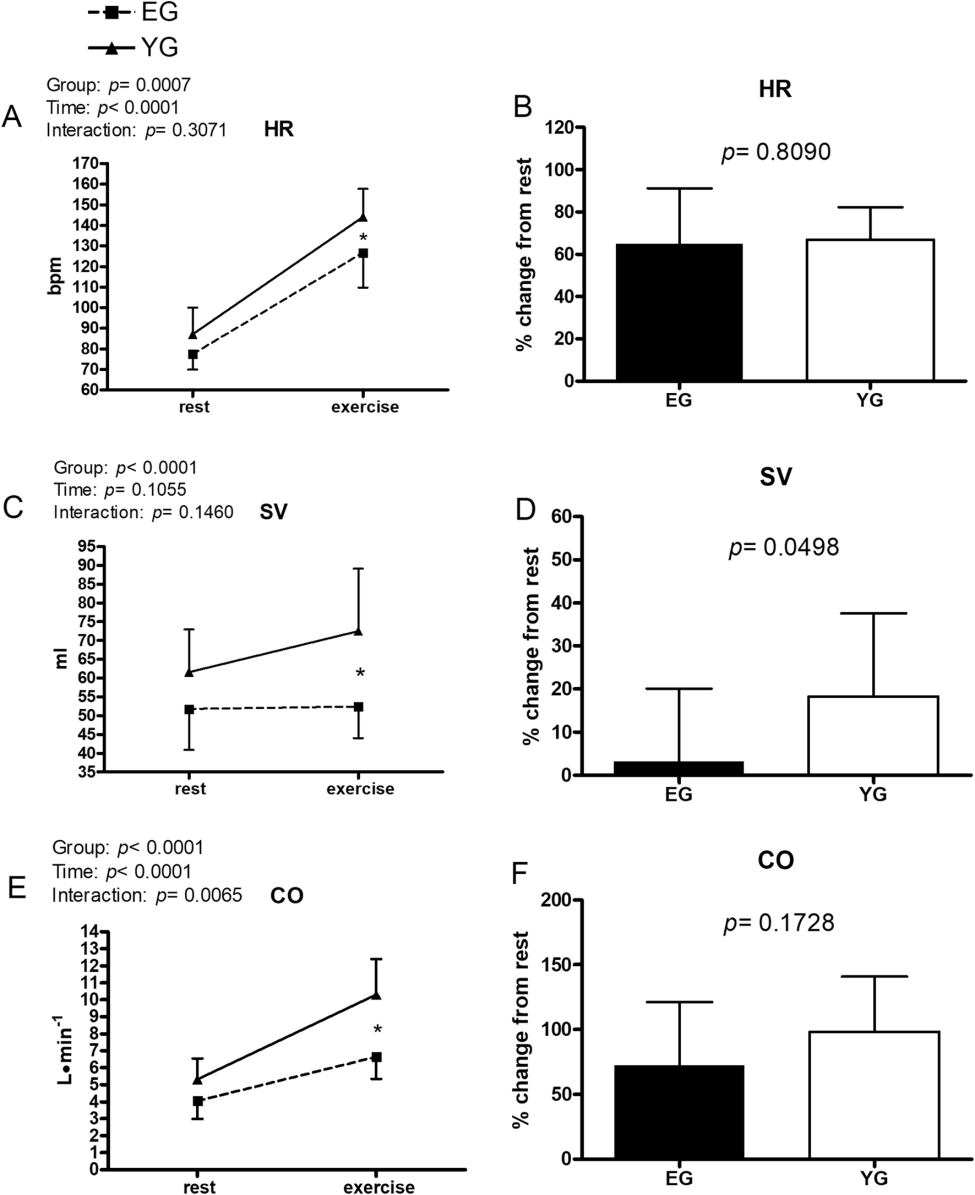
Hemodynamic data at rest and during exercise conducted at the gas exchange threshold intensity in the elderly (EG, n = 11) and in young (EG, n = 13) groups. Panels (**A**,**C**,**E**) show absolute values; panels (**B**,**D**,**F**) show % changes from rest. *HR* heart rate, *SV* stroke volume, *CO* cardiac output. Values are mean ± SD. *p < 0.05 between groups at the same timepoint.

**Figure 2:**
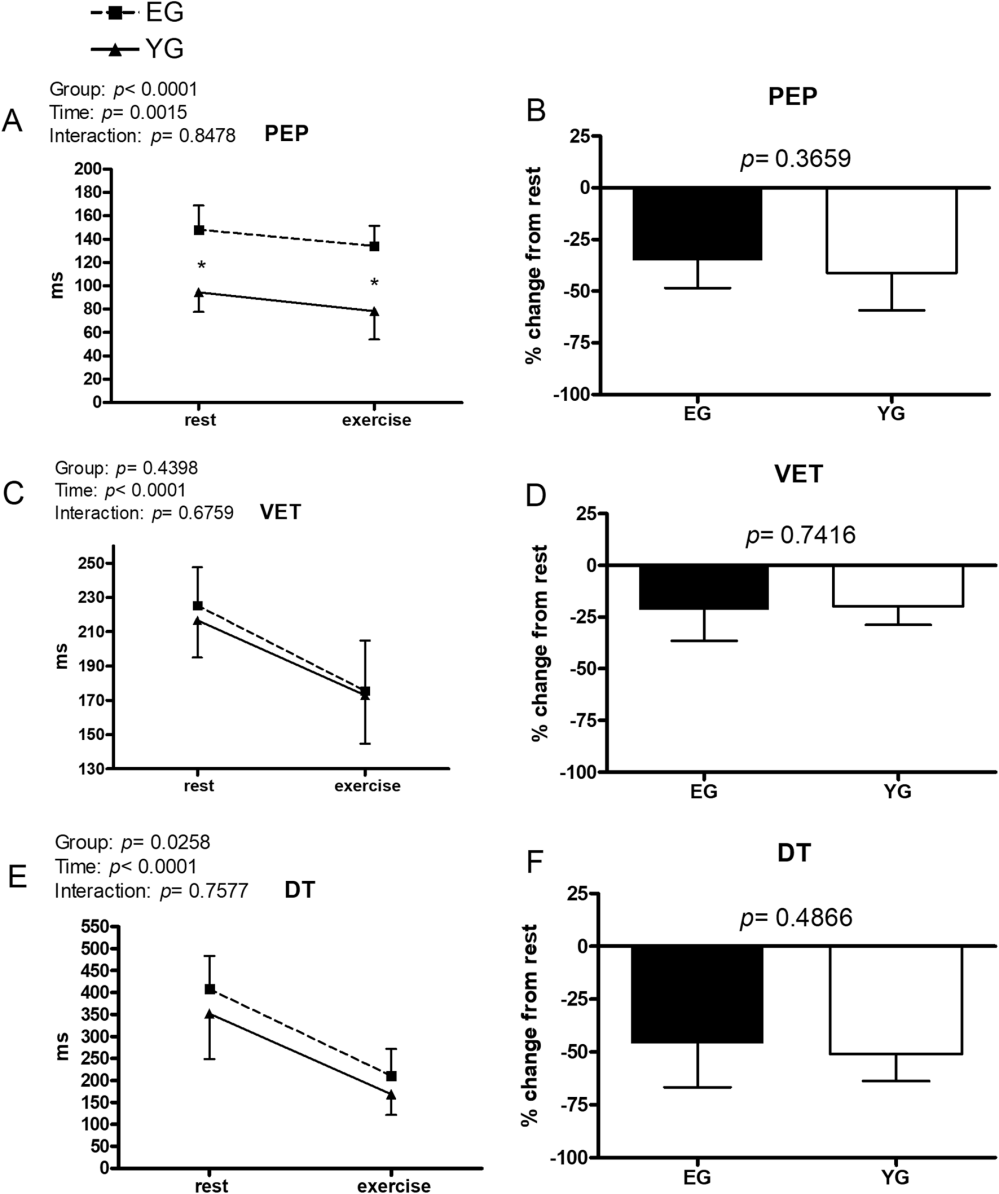
Hemodynamic data at rest and during exercise conducted at the gas exchange threshold intensity in the elderly (EG, n = 11) and in young (EG, n = 13) groups. Panels (**A**,**C**,**E**) show absolute values; panels (**B**,**D**,**F**) show % changes from rest. *PEP* pre-ejection period, *VET* ventricular ejection time, *DT* diastolic time. Values are mean ± SD. * = p < 0.05 between groups at the same timepoint.

**Figure 3:**
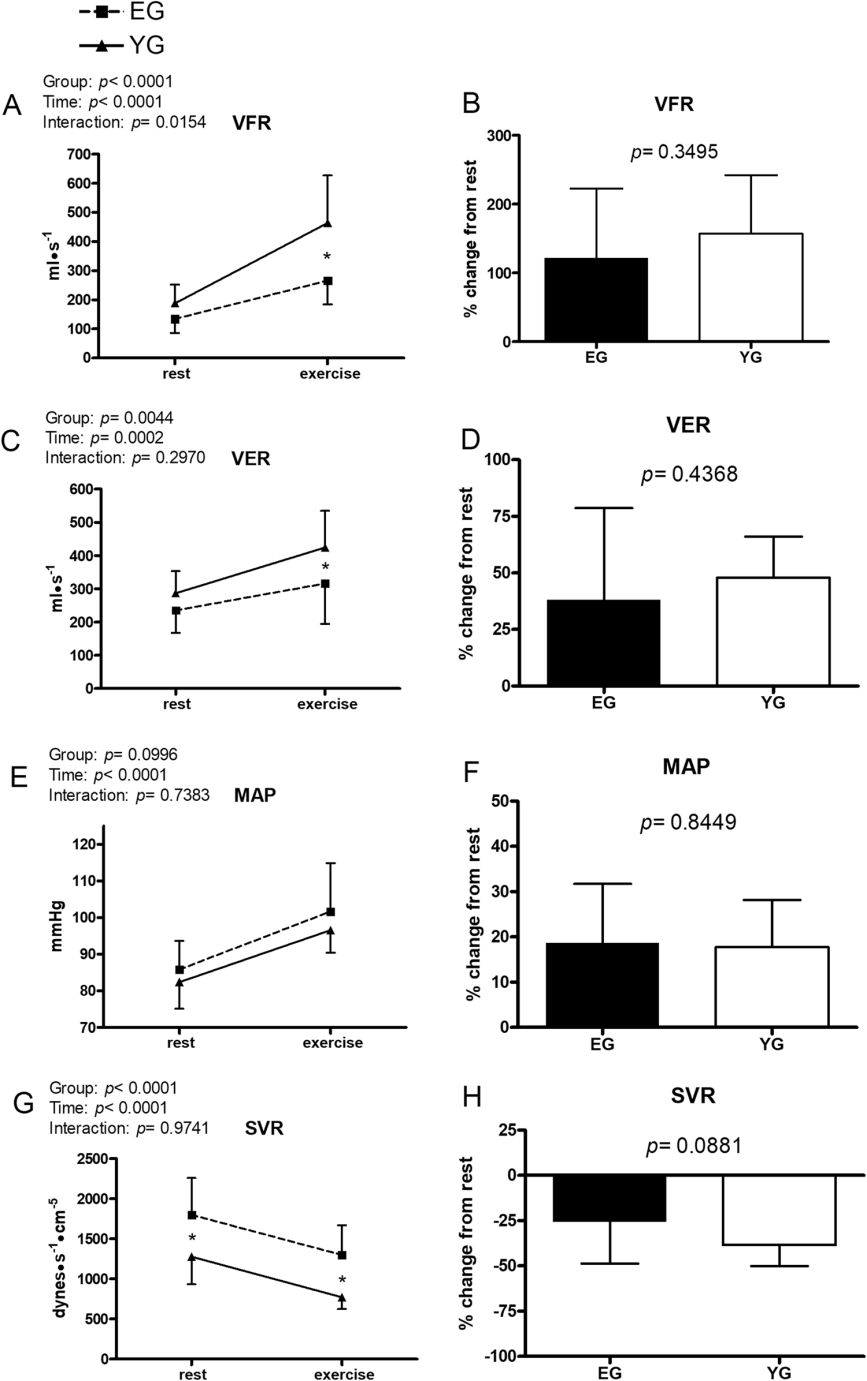
Hemodynamic data at rest and during exercise conducted at the gas exchange threshold intensity in the elderly (EG, n = 11) and in young (EG, n = 13) groups. Panels (**A**,**C**,**E**,**G**) show absolute values; panels (**B**,**D**,**F**,**H**) show % changes from rest. *VFR* ventricular filling rate, *VER* ventricular emptying rate, *MAP* mean arterial pressure, *SVR* systemic vascular resistance. Values are mean ± SD. *p < 0.05 between groups at the same timepoint.

**Figure 4:**
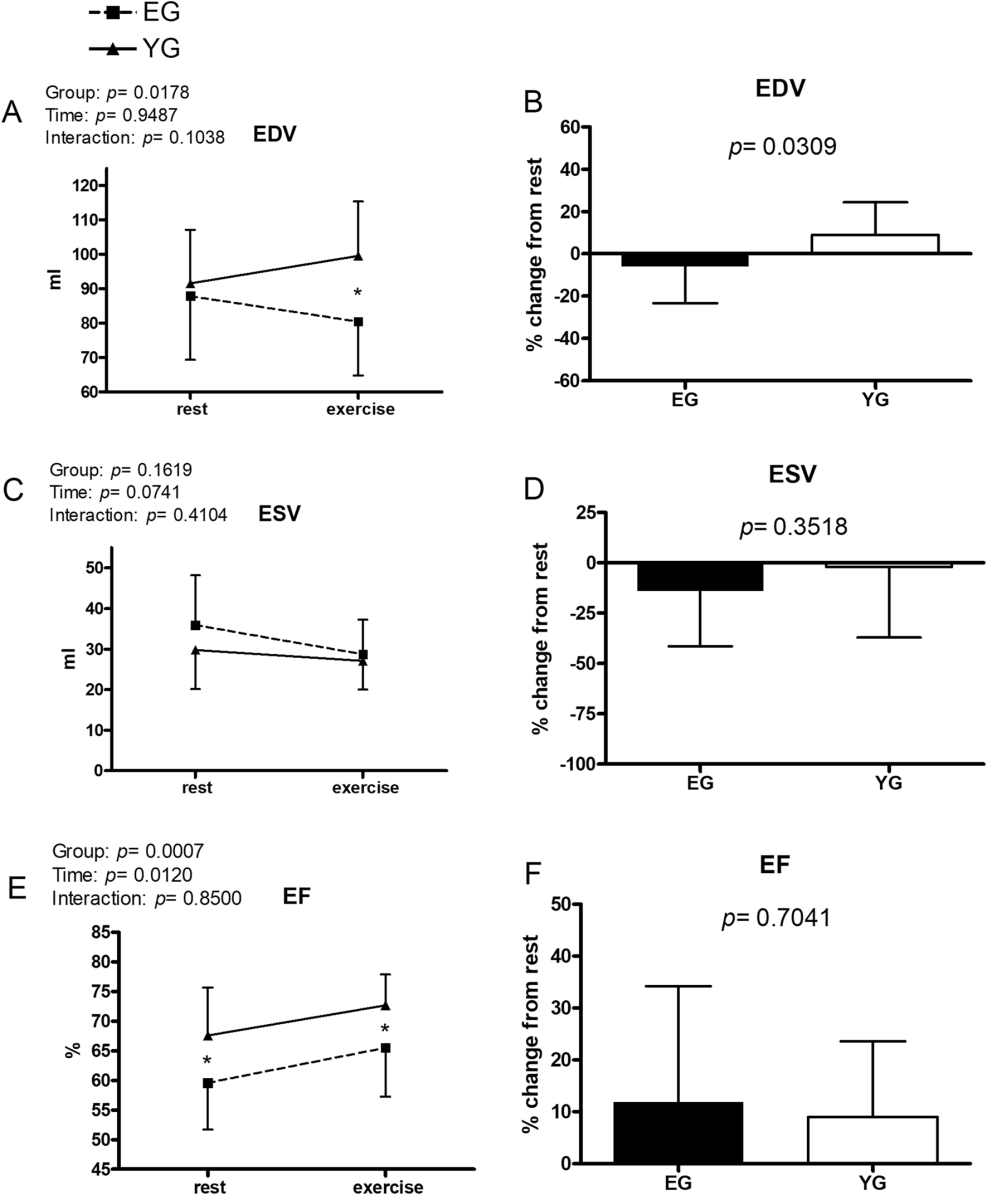
Hemodynamic data at rest and during exercise conducted at the gas exchange threshold intensity in the elderly (EG, n = 11) and in young (EG, n = 13) groups. Panels (**A**,**C**,**E**) show absolute values; panels (**B**,**D**,**F**) show % changes from rest. *EDV* end-diastolic volume, *ESV* end-systolic volume, *EF* ejection fraction. Values are mean ± SD. *p < 0.05 between groups at the same timepoint.

**Figure 5:**
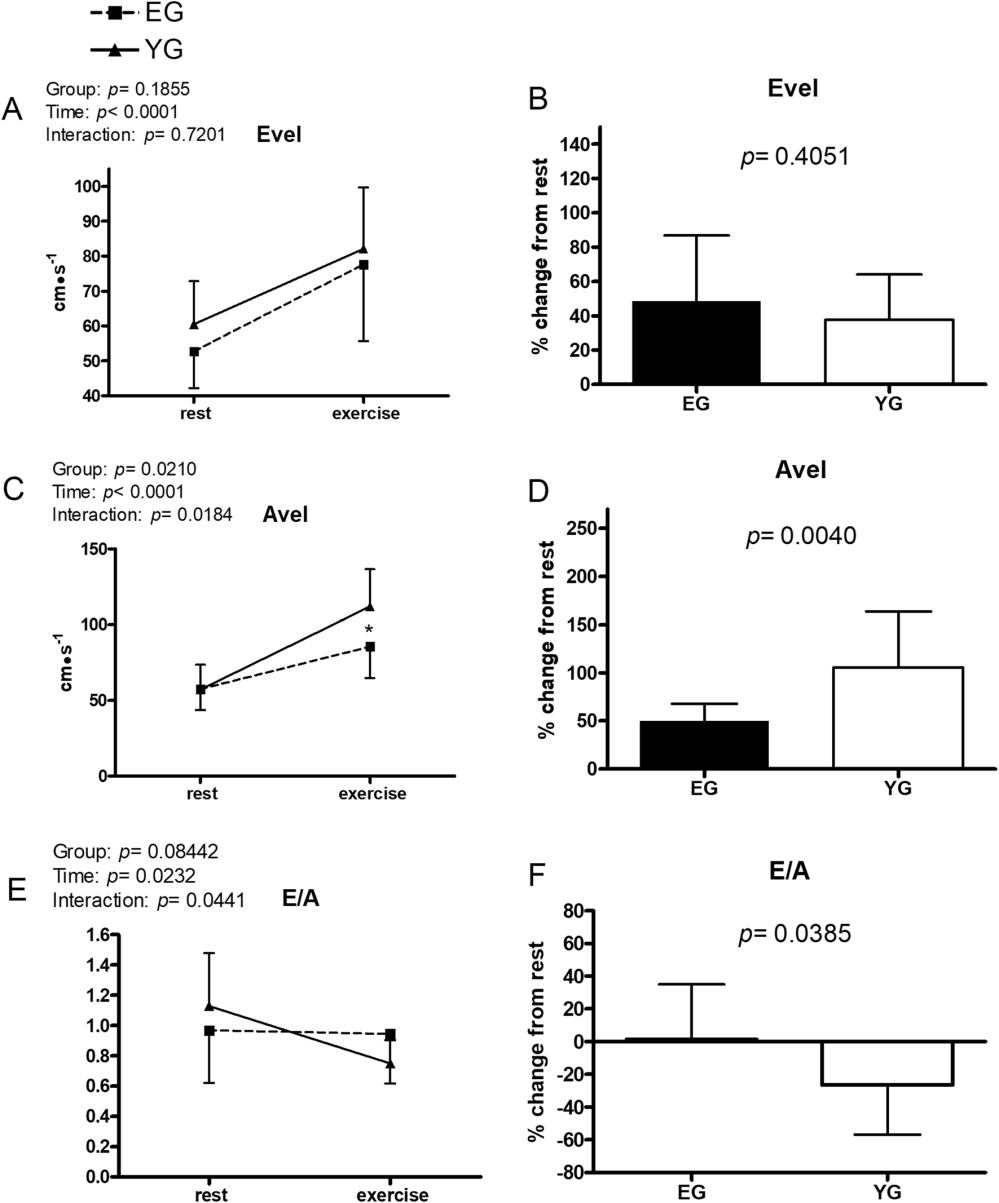
Hemodynamic data at rest and during exercise conducted at the gas exchange threshold intensity in the elderly (EG, n = 11) and in young (EG, n = 13) groups. Panels (**A**,**C**,**E**) show absolute values; panels (**B**,**D**,**F**) show % changes from rest. *Evel* transmitral filling peak velocity during early diastole, *Avel* transmitral filling peak velocity during atrial contraction. Values are mean ± SD. *p < 0.05 between groups at the same timepoint.

**Figure 6:**
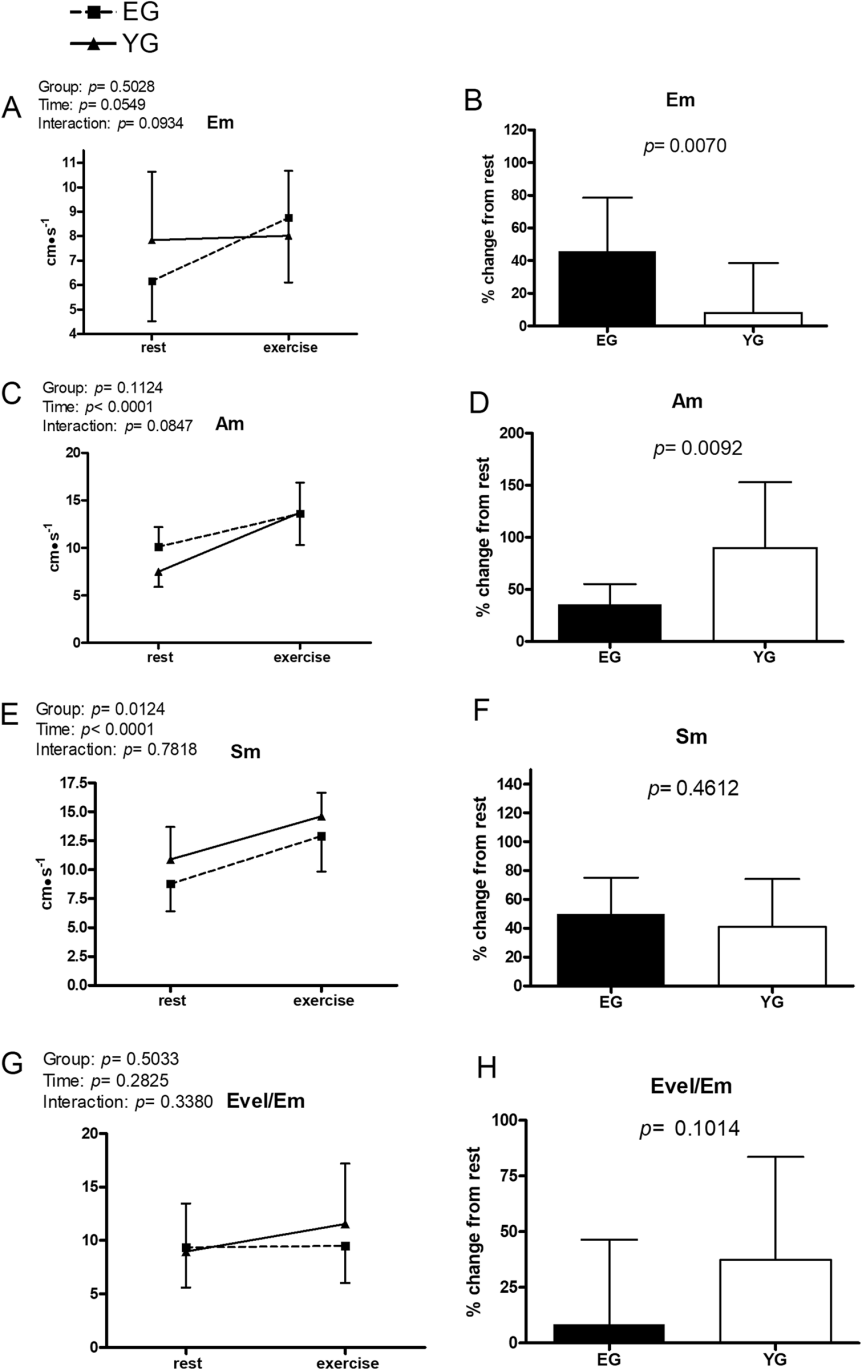
Hemodynamic (tissue Doppler) data at rest and during exercise conducted at the gas exchange threshold intensity in the elderly (EG, n = 11) and in young (EG, n = 13) groups. Panels (**A**,**C**,**E**,**G**) show absolute values; panels (**B**,**D**,**F**,**H**) show % changes from rest. *Em* mitral valve motion velocity during early transmitral filling, *Am* mitral valve motion velocity during atrial contraction, *Sm* systolic myocardial tissue velocity at mitral anulus. Values are mean ± SD. *p < 0.05 between groups at the same timepoint.

Figures 1, 2, 3, 4, 5 and 6 exhibit data collected at rest and at the fifth minute of exercise conducted at GET intensity. In addition, the % changes of each cardiovascular parameter with respect to rest are presented.

Figure 1 demonstrates that HR increased during the GET test with respect to rest, with the YG reaching a higher HR level as compared to the EG (panel A). HR was 144.1 ± 13.6 vs. 126.7 ± 16.9 bpm for the YG and the EG, respectively. It is noteworthy that these HR values were close to those reached at the GET workload during the preliminary CPT (see Table 1). Panel B of Fig. 1 illustrates that the HR % increment with respect to rest was not different between groups. The YG had a higher SV during the GET test in comparison with the EG (Fig. 1, panel C). SV was 72.5 ± 16.7 vs. 52.4 ± 8.4 ml for the YG and the EG, respectively. The SV % increment with respect to rest was also higher in the YG than in the EG, reaching statistical significance (panel D). As a result of the higher HR and the SV increments in the YG, the YG also achieved a higher CO level in comparison with the EG (panel E), although the % increment with respect to baseline was not different between groups (panel F).

Figure 2 (panel A) shows that (pre-ejection period) PEP was shorter in the YG than in the EG both at rest (94.5 ± 17.0 vs. 148.0 ± 20.9 ms; YG and EG, respectively) and during exercise (78.4 ± 24.3 vs. 134.1 ± 17.3 ms; YG and EG, respectively). However, there was no difference between the groups in the capacity to shorten PEP as illustrated by the % decrement with respect to rest (panel B). In both groups, ventricular ejection time (VET) was on average lower during exercise with respect to rest, and there was no difference between groups neither in the absolute values of VET (panel C) nor in its % change from rest (panel D). Diastolic time (DT) demonstrated a significant group effect (panel E), and it was on average shorter in the YG as compared to the EG. However, post-hoc comparison between columns did not find any significant difference at rest as well as during exercise. Panel F demonstrates that % DT shortening due to exercise was similar between groups.

Figure 3 shows that the YG had a higher ventricular filling rate (VFR) in response to exercise as compared to the EG (463.9 ± 162.8 vs. 265.9 ± 81.6 ml·s^−1^, YG and EG, respectively; panel A), although no difference between groups was found in the capacity to increase this parameter in percent from rest (panel B). Similarly, the YG had a higher ventricular emptying rate (VER) in response to exercise as compared to the EG, which reached a value of 424.5 ± 110.3 vs. 316.1 ± 121.3 ml·s^−1^ in the YG and in the EG, respectively (panel C). No difference was found between groups in the % VER increment from rest (panel D). Aging did not influence the mean arterial blood pressure (MAP) level both at rest and during exercise (panel E), nor its % increment from rest (panel F). In contrast, systemic vascular resistance (SVR) was significantly higher in the EG as compared to the YG both at rest (1798.7 ± 458.1 vs. 1277.4 ± 345.1 dynes·s^−1^·cm^−5^, for the EG and the YG, respectively) and during exercise (1299.0 ± 369.8 vs. 771.3 ± 147.7 dynes·s^−1^·cm^−5^, EG and YG, respectively; panel G). The capacity to decrease SVR during exercise from rest was not different between groups (panel H).

Panel A of Fig. 4 shows that EDV was similar between groups at rest. However, during exercise, EDV was significantly higher in the YG than in the EG (99.5 ± 15.8 vs. 80.4 ± 15.6 ml, YG and EG, respectively). Moreover, the % increase of EDV in response to exercise was greater in the YG as compared to the EG (panel B). No difference was discovered between groups neither in the ESV level, nor in its % increment from rest (panel C and D, respectively). EF (panel E) was on average lower in the EG than in the YG both at rest (59.6 ± 7.9 vs. 67.6 ± 8.1%, EG and the YG, respectively) and during exercise (65.5 ± 8.2 vs. 72.7 ± 5.2%, EG and the YG, respectively). In both groups, EF increased to a similar extent with respect to rest (panel F).

In both groups, exercise significantly increased early transmitral filling peak velocity (Evel) with respect to rest (Fig. 5, panel A), without any detectable difference in each group’s % increase of this parameter with respect to rest (panel B). Avel (panel C) also increased significantly during exercise from rest, with the YG showing a higher increase in this parameter than the EG (112.0 ± 24.5 vs. 85.5 ± 20.7 cm·s^−1^, YG and EG, respectively). Furthermore, the YG exhibited a greater percent enhancement in atrial transmitral filling peak velocity (Avel) than the EG (panel D). No difference was found between groups with regards to the ratio between early and atrial transmitral filling peak velocities (E/A; panel E), however YG decreased this parameter in response to exercise, while the EG did not.

Figure 6 illustrates variables gathered with tissue Doppler. While the peak velocity of mitral valve motion during early diastole (Em) remained unchanged in the YG and increased in the EG with exercise, on average Em was not significantly affected by exercise (panel A). However, while there was no significant difference between groups, the EG showed a higher % increment with respect to rest than the YG (panel B). In both groups, the peak velocity of mitral valve motion during late diastole (Am) increased with respect to rest in response to exercise (panel C), although the % increase in this parameter was higher in the YG as compared to the EG (panel D). Systolic myocardial velocity (Sm) was on average higher in the YG than in the EG, although post-hoc analysis did not yield any significance at rest or during exercise. Moreover, in both groups, this parameter significantly increased in response to exercise (panel E). There was not a significant difference between groups in the Sm % change from rest (panel F). Time and group did not affect the ratio between the early transmitral filling peak velocity and the peak velocity of mitral valve (Evel/Em; panel G), nor any difference was found between groups in the % change from rest (panel H).

## Discussion

Exercise is a powerful tool to investigate how the cardiovascular system responds to stress and to assess its functional reserves. The importance of assessing the circulatory adjustments to exercise as a marker of cardiovascular health is well-acknowledged^1^. Moreover, information about expected values may aid in establishing targets and providing parameters to evaluate the impact of training interventions. This is particularly important in the elderly, as cardiovascular, metabolic, and respiratory diseases often develop with aging, and these functional reserves deteriorate. The present study was conducted to compare hemodynamics of healthy elderly and young individuals during an effort conducted at the same relative submaximal workload, i.e., at the GET intensity. This workload discriminates the transition from moderate to heavy exercise and identifies a workload that can be sustained for long periods^25^. To the best of our knowledge, very little information exists about the hemodynamic differences between young and elderly individuals during exercise at submaximal intensities. It should be considered that, rather than maximal workloads, submaximal exercise protocols are normally employed to optimise training and to build cardiovascular and pulmonary rehabilitative programs in the clinical setting. Sub-maximal workloads allow longer sessions of physical activity and higher training volumes than training at heavier workloads and produces favourable adaptations both at cardiovascular and at muscle level, thereby improving exercise tolerance. In this context, the identification of the GET is paramount as it discriminates the transition from moderate to heavy exercise^25^. Moreover, most of the previous conducted to compare hemodynamics during exercise between young and elderly individuals has used exercise up to maximum workloads, which leads to very different absolute workloads, VO_2_, and HR levels. Thus, this approach has an intrinsic limit. Alternatively, other investigators used % of maximum HR to set the workload. However, even this approach has its own limits as there is substantial difference among individuals in the HR response to exercise, i.e., every subject has her/his individual HR response at a given intensity of exercise. Therefore, taking into consideration these limits, we chose to conduct experiments at the GET intensity, a submaximal workload that marks the transition from mild to moderate exercise intensity, that can be tolerated for minutes, that allows a steady state in hemodynamic parameters, and that produces a similar metabolic response among subjects^25^.

Our main hypothesis was that elderly individuals would show a reduced capacity to increase SV and CO as compared to young individuals. Specifically, we hypothesised that reduction in cardiac inotropism as well as in diastolic functions impaired the SV response at submaximal intensity, thereby explaining at least in part the reduced capacity to exercise of the EG as compared to the YG. Our results confirmed this hypothesis, as the EG showed a reduced capacity to increase SV in response to exercise in comparison with the YG (see Fig. 1, panel C). This phenomenon was previously described in investigations with incremental exercise tests up to exhaustion^9,14^, however, to the best of our knowledge, the present study is the first to compare hemodynamics during submaximal rectangular exercise at the same relative intensity. Moreover, our results show that not only the EG did have a reduced absolute SV response, but their capacity to increase SV with respect to rest was blunted in comparison with the YG as well (see Fig. 1, panel D), thereby reinforcing our hypothesis of an impaired capacity to properly increase SV during effort in the elderly.

The reduced SV, in combination with the lower HR, resulted in a lower CO in the EG than in the YG, thereby explaining, at least in part, the lower workloads achieved by the EG at the GET. It is noteworthy that in the EG, although the absolute HR was lower, the chronotropic response was increased to the same relative extent as in the YG, as illustrated by the similar % increase in HR with respect to rest in both groups (see Fig. 1, panel B). Furthermore, in the EG, HR was on average 126 bpm during exercise, which is approximately 86% of the maximum HR reached in their preliminary CPT (see Table 1). Similarly, at the GET, the YG reached a HR of 144 bpm, which was on average approximately 81% of the maximum HR achieved during their preliminary CPT. Taken together, these findings suggest that in regard to chronotropic reserve, the two groups exercised at a similar relative intensity, thereby reinforcing the use of the GET as a useful tool to compare exercise intensities at submaximal intensities. Is it also worth noting that the lower absolute HR in the EG was not due to chronotropic incompetence per se (same % increase), rather it was due to the reduction on HR_max_ which usually accompanies normal aging.

The impaired capacity to increase SV during the GET test in the EG likely resulted from the convergence of several concomitant phenomena: a reduced capacity to increase cardiac pre-load, a reduced capacity to augment cardiac contractility, and an increase in vascular resistance.

The EG demonstrated an impaired capacity to increase cardiac pre-load as demonstrated by their EDV behaviour during exercise. At rest, EDV was similar between groups, however the YG was able to increase EDV in response to exercise, while individuals of the EG were not. Moreover, the EG also exhibited a reduced % increase in EDV from rest (Fig. 4, panel B). Therefore, these findings suggest that aging is accompanied by an impairment in the capacity to enhance pre-load in response to exercise. This is line with the evidence that diastolic function deteriorates with age, likely due to an increase in cardiac stiffness and a decrease in ventricular relaxation^16–20^. Moreover, VFR, a measure of diastolic flux, was more elevated in the YG than in the EG during exercise, reinforcing the hypothesis that elderly people cannot properly enhance cardiac flux during diastole. In this regard, it should be pointed out that DT was on average longer in the EG as compared to the YG, and this should have permitted a more efficient cardiac filling in the EG; however, this was not the case as previously discussed. Collectively, the EDV and VFR data indicate that in the elderly preload reserve cannot be recruited to the same extent as in younger individuals during submaximal exercise. This is also in line with previous findings in healthy men reporting that the reduced diastolic filling during exercise that occurs with aging is likely due to structural changes which accompany normal aging^27^.

The second phenomenon which could have responsible for the blunted SV response in the EG was myocardial contractility. The YG had a higher resting EF when compared to the EG. This difference in EF between groups remained during exercise. However, the contractility reserve was similarly recruited in the two groups, as EF increased to the same relative extent during exercise from rest (see Fig. 4, panel F). It has previously been observed that the EF response is reduced in the elderly, and it is speculated that this impaired myocardial performance is due to a combination of an increased SVR, a decreased effectiveness of the autonomic nervous system to modulate myocardial contractility, and an impaired intrinsic cardiomyocyte contractility^1,9,14^.

We found that PEP was longer in the EG than in the YG. PEP is inversely related to the development of intraventricular pressure as well as cardiac sympathetic activity^28^. The longer PEP in the EG than in the YG reinforces the hypothesis that myocardial contractility was reduced in the EG at rest and during exercise. However, it is important to note that PEP decreased to a similar extent in both groups, suggesting that cardiac sympathetic stimulation could still effectively recruit some reserve of contractility in the EG. To further support our hypothesis that myocardial contractility could be still recruited in the EG there is Sm, which showed a behaviour similar to that of EF and PEP, i.e., it was on average lower throughout tests in the EG than in the YG. However, during exercise, Sm increased to a similar extent between groups in term of percent increase from rest. Sm is a parameter gathered with tissue Doppler, and it is considered an index of contractility as it is correlated with ventricular peak dP/dt and is reduced in patients with ventricular dysfunction^29–32^. The contention that sympathetic stimulation was able to effectively recruit contractility in the EG is also supported by VET, which shortens when contractility increase^28^. VET was similar between groups at rest, and it decreased to a similar extent in in both groups in response to exercise. Moreover, both groups exhibited a similar capacity to reduce ESV during exercise, further reinforcing the hypothesis that the EG still had the capacity to recruit their contractility reserve.

An alternative explanation for the reduced EF in the EG may be their higher SVR. In this regard, it is useful to consider the VER behaviour, which represents the ventricular emptying capacity, and it is considered an index of myocardial performance^33,34^. The EG was unable to properly enhance VER during exercise as compared to the YG, notwithstanding the two groups had the same VET. This could be the consequence of the higher level of SVR in the EG, which potentially limited ventricular emptying in comparison with YG. This result is in line with the fact that older individuals have higher levels of SVR, both at rest and during exercise, thus suggesting that vascular resistance is increased by aging. This fact may, at least in part, explain the reduced EF and ventricular emptying observed in the EG. It should be considered that healthy aging is associated with an elevation in vascular tone in the muscle, both at rest and during exercise^35,36^. Inasmuch as adequate muscle perfusion is vital to meet the metabolic demand of the tissue, enhanced vascular tone is an important potential limitation of the capacity for physical activity. Thus, the reduced workload we observed in the EG may be also the consequence of reduced muscle perfusion secondary to enhanced vascular tone.

Our data suggest that heathy aging did not significantly impact the capacity to increase MAP during exercise, and this indicates that mechanisms controlling blood pressure during exercise were well-preserved in the EG.

Concerning the parameters gathered by transmitral and tissue Doppler, Evel was not influenced by age neither at rest nor during exercise. Moreover, the capacity to increase Evel in response to exercise was preserved in the EG, as illustrated in panel B of Fig. 5. Similar results were observed for Em; however, it appears that in the EG, the % increase of Em from rest was more pronounced than in the YG. Taken together, these findings appear to contradict the notion that healthy aging is associated with an increase in cardiac stiffness and/or a decrease in ventricular relaxation capable of impairing early ventricular filling^16,19,37^. However, other observations suggest that ventricular filling was actually altered in the EG. Specifically, the E/A < 1 observed in older individuals at rest suggests an impaired left ventricular relaxation, implying that a more vigorous atrial contraction was required to maintain or increase left ventricular filling. It is therefore conceivable that individuals in the EG were not able to further increase left ventricular filling through a more vigorous atrial contraction during exercise, unlike the YG. Furthermore, although diastolic time was similar between groups, HR was lower in the EG. In this regard, it should be considered that increasing HR reduces diastolic filling time, so that diastolic time would have been even shorter in the EG at greater heart rates, and this may have reduced further the cardiac preload. Thus, our results suggest that an impaired ventricular relaxation was present in older individuals, which likely contributed to the lower EDV and SV. In short, our finding that the EG was not able to recruit the reserve of atrial contraction to enhance ventricular filling may be considered an early sign of diastolic stiffening of the left ventricle.

It is also important to consider that our EG was composed of physically active individuals, and this may explain our observations of relatively well-preserved hemodynamic capacities in the older group, as SV was indeed well preserved. Concerning the YG, our findings are instead consistent with previous investigations reporting that Evel and Em do not show any relationship with SV during exercise in healthy young individuals^21,38^. Therefore, this suggests that a faster early filling velocity is not the primary mechanism that improves ventricular filling in young individuals during submaximal workloads, such as those employed in the present investigation.

To fully understand the complexity of diastolic filling, however, the contribution of the atria must also be considered. In this regard, Avel increased in both groups in response to exercise, but the increment was more evident in the YG than in the EG. This difference between groups was demonstrated by both absolute Avel values and by the Avel % increment with respect to rest. These findings suggest that the YG relied more on the capacity to increase the atrial contribution to ventricular filling than the EG. Supporting this hypothesis, the % increment with respect to rest of Am was more elevated in the YG as compared to the EG. These different Avel and Am behaviours between groups can be explained by an increased ventricular stiffness and/or an impaired atrial contraction in the EG as compared to the YG. The present experimental set-up precludes an explanation of whether ventricular stiffness and/or impaired atrial contraction were both present in the EG. However, whatever the cause, it appears as though the YG relied more on atrial reserve than the EG to fill the ventricle during submaximal exercise. It should also be considered that Avel and Am increase with increments in HR in healthy subjects^39,40^, thus it is possible that increasing HR could have resulted in an increase in atrial contribution to ventricular filling in the EG. However, we believe that this occurrence was unlikely given that the EG group exercised at a lower absolute HR (i.e., on average about 126 vs. 144 bpm), but at a higher HR reserve than the YG. More specifically, estimating the HR reserve as 220-age^41^, the EG exercised at about the 87% of their chronotropic reserve, while the YG at about 75%. The Evel and Avel % increases from rest in the EG were similar. In contrast, the Avel increment was steeper than Evel in the YG, and therefore the E/A ratio decreased more in the YG than in the EG. These findings further support the hypothesis that in the YG the capacity to enhance ventricular filling during submaximal exercise relies more on the recruitment of the atrial contribution than on an increase in early ventricular filling. This is in line with recent findings demonstrating that during effort, when the cardiac cycle shortens due to tachycardia, atrial contraction becomes determinant in left ventricular filling^42^. Specifically, it was reported in endurance athletes that the left atrium plays an important role in maintaining and increasing EDV when filling is compromised, such as during tachycardia^40^. In this regard, it should also be mentioned that previous research reported that the E/A gradually decreases with increasing exercise intensity in young individuals, and this has been interpreted as a more prominent increase in atrial vs. early ventricular filling during exercise^43^. This result is also in line with another investigation reporting a larger decrement in E/A in young vs. old subjects during exercise^21^. Moreover, in older male athletes it was observed that SV is not related to faster filling during early or late ventricular filling^21^.

Evel/Em is the ratio between the maximum velocity of early rapid filling and the maximum velocity of mitral valve anulus during early filling and is considered a load-independent estimate of end diastolic pressure^44^. Although this measure has been questioned in the clinical setting^45^, the fact that in our experimental setting Evel/Em did not differ between groups suggests that ventricular filling pressure is unaffected by aging during exercise at the GET intensity. This result is in contrast with previous observations reporting that aging is associated with an increased pulmonary artery pressure during exercise, which develops as the consequence of both increased pulmonary vasculature resistance and higher left ventricular filling pressures^37,46^. As previously exposed, it should be considered that in our investigation the EG was composed of physically active individuals, and this may explain at least in part the different outcome from previous studies. Moreover, difference in exercise modalities and methods of measurements exist between the present and previous investigations, and this may account for the different outcomes.

One potential limitation of the present investigation is that we studied physically active, healthy individuals. Therefore, our results cannot be applied to non-active, elderly individuals, or those suffering from any cardiovascular, metabolic, or respiratory disease, which are, incidentally, more common as a result of non-active aging^47^. Further research conducted with in-active elderly individuals and/or individuals suffering from age-related diseases is warranted to gather a clearer picture of the hemodynamic consequences of aging in these populations. A further potential limit is that the E and A waves of the transmitral Doppler may merge during exercise-induced tachycardia^48^. Thus, the increase in Avel observed in both groups during exercise may have been affected by a contribution from the E wave.

In conclusion, our data suggest that healthy aging is characterized by several concomitant changes in hemodynamics during submaximal exercise at the GET intensity with respect to young people. The well-known reduction in chronotropism is accompanied by a reduced capacity to increase SV, which is a major finding of the present investigation. This is the result of several complex phenomena, namely impaired ventricular filling rate and ventricular relaxation, a reduced capacity to increase EDV, an impaired contractile response, and an elevation in systemic vascular resistance. In short, all the main hemodynamic modulators, i.e., chronotropism, contractility, cardiac pre-load, and vascular resistance, are significantly affected by normal aging. One new finding of the present investigation is the importance of atrial contraction on ventricular filling, as it appears that the atrial reserve can be recruited by young individuals to increase EDV during submaximal exercise, but not the elderly. Further research is warranted to better clarify this latter point.

## Methods

### Subjects

Two groups of subjects were studied:

1. The EG was composed of 11 healthy individuals (7 females and 4 males) older than 60 years [range of age 60–72 years; mean ± SD 65.18 ± 4.71]. At the time of the study, based on their reports, all were physically active accordingly to the World Health Organization recommendations (https://www.who.int/news-room/fact-sheets/detail/physical-activity) and were practicing Tai Chi Chuan, three times a week, for an average of 6 years.
2. The YG was composed of 13 healthy individuals (7 females and 6 males) younger than 35 years (range of age 18–35 years; mean ± SD 29.84 ± 4.06). At the time of the study, all were physically active and were regularly involved in exercise activities in a gym for at least three times a week.

In both groups, recruitment was conducted after a medical visit to exclude the presence of cardiac, pulmonary, and metabolic conditions. Subjects under medications for any known disease were excluded. Smoking was also considered as exclusion criterion. We decided to enroll physically active individuals since, especially with aging, inactivity is often linked to cardiovascular and metabolic diseases. Thus, physically active people should be considered as the “normality” either in youth or in aging^49^. We specifically enrolled Tai Chi Chuan practitioners for the EG as elderly individuals that practice this discipline have demonstrated the ability to maintain good nutritional status, body composition, and muscle functionality^50^.

To calculate the required sample size, we used a calculator freely available on the internet (https://clincalc.com/stats/samplesize.aspx). The criteria set to calculate the sample size were: (1) a power of 85%, (2) an overall type 1 error of 0.05, (3) a SD of 20%, and (4) a 25% difference between groups in the studied variables, specifically SV and CO. Ten subjects/group were required to obtain adequate statistical power.

The research was approved by the Independent Ethical Committee of the A.O.U. of Cagliari (PG/2017/1700). Each participant was informed about the purposes and methods of the research and signed consent to participate in the investigation, which was carried out in accordance with the Declaration of Helsinki.

### Experimental protocol

The experimental protocol began with a preliminary cardiopulmonary CPT to establish the workload corresponding to that of the GET. Subjects were asked to abstain from drinking alcohol or coffee for at least 24 h before scheduled tests. All experiments were conducted in a room with controlled temperature and humidity (22 °C; 50% relative humidity).

### Cardiopulmonary test

Each subject underwent a CPT on an electromagnetically braked cycle-ergometer (CUSTO Med, Ottobrunn, Germany). ${\dot{\text{V}}}$O_2_, ${\dot{\text{V}}}$CO_2_, and V_E_ data were collected breath by breath with a gas analyzer (Ultima CPX, MedGraphics St. Paul, MN, USA). RER was calculated as ${\dot{\text{V}}}$CO_2_/${\dot{\text{V}}}$O_2_. The gas analyzer was calibrated immediately before the CPT, as indicated by the manufacturer. The exercise consisted of a linear increase in workload (20 or 30 W·min^−1^ for the EG and YG respectively, starting at 20 or 30 W) while maintaining a pedaling frequency of 60 rpm until exhaustion (the point at which the subject was unable to maintain a pedaling rate of at least 50 rpm). W_max_, ${\dot{\text{V}}}$O_2max_, (${\dot{\text{V}}}$CO_2max_), maximum ventilation (V_Emax_), and HR_max_ were calculated as the average of the last 15 s of exercise. Subjects were considered to reach their ${\dot{\text{V}}}$O_2max_ when they met at least two of the following criteria: (1) a plateau in ${\dot{\text{V}}}$O_2_, despite an increasing workload (< 80 ml·min^−1^), (2) RER above 1.10, and (3) HR ± 10 beats·min-1 of predicted maximum HR, calculated as 220-age^51^.

The GET was calculated using the V-slope method, which detects the exchange threshold by employing a regression analysis of the slope of ${\dot{\text{V}}}$CO_2_ plotted as a function of ${\dot{\text{V}}}$O_2_^52^. During the CPT, subjects were familiarized with the equipment and the staff of the laboratory, thereby allowing habituation to the environment and the ergometer that was employed in the successive experimental session. Throughout the CPT, participants were also monitored with ECG to exclude any cardiovascular problem induced by exertion.

### Sessions to study hemodynamics during exercise at GET intensity

After the CPT session (interval 4–7 days), each participant reported to the laboratory and performed the GET test: a rectangular exercise session pedaling on the same cycle-ergometer utilized for the CPT. Before the subjects began the GET test, they sat on the cycle-ergometer for three minutes to collect data at rest. The exercise consisted of five minutes pedaling against a workload corresponding to that of the GET previously measured during the CPT. A recovery of six minutes was allowed.

### Hemodynamic measurements

Hemodynamics were measured using an echocardiographic machine equipped with a hand-held 3.5-MHz adult ultrasound probe (Vivid iq, GE Healthcare, Fairefield, CT, USA). HR was assessed as the reciprocal of the electrocardiogram R-R interval provided by the echocardiograph. Two dimensional images and pulsed Doppler recordings were acquired in the sitting position using the apical four-chamber view. Left ventricular ESV and EDV were calculated automatically by the software using the conventional formula 8A2/3πL, where A is the left ventricular area and L is the longest ventricular length^53^. Ventricular area was determined by tracing along the inner edge of the endocardium and ventricular length was considered as the distance from the ventricular apex to the midpoint of the mitral annulus. Left ventricular EF was calculated as: (EDV-ESV/EDV) ·100.

SV was calculated as EDV-ESV, and CO as SV · HR.

During the same beats utilized for ESV and EDV measurements, Evel, Avel, and E/A were measured with pulsed-wave Doppler (PWD), with a 3-mm PWD sample volume placed distal to the mitral anulus, between the mitral leaflets. The interrogation beam was aligned with mitral flow^54^.

Em and Am were assessed with Doppler tissue imaging, with images captured from the apical four-chamber view. The pulsed-wave sample volume was placed at the lateral mitral anulus. The ratio Evel/Em was considered as an estimate of left ventricular filling pressure^30,55^. Sm was also assessed to obtain a measure of longitudinal systolic function^30^. Since tissue Doppler measures are highly dependent on the angle between the scan beam and the vector of ventricular motion^56^, particular care was taken with these measures.

Aortic valve Doppler sampling was also carried out from the apical five-chamber window to assess PEP and VET. In detail, PEP was measured as the time from the beginning of the QRS complex of the electrocardiogram and the opening of the aortic valve, and VET was measured as the total duration of the ejection period in the Doppler trace. DT was calculated by subtracting the sum of PEP and VET from the total period of the cardiac cycle.

The ratio between SV and DT was calculated to obtain a measure of the mean rate of diastolic blood flow, i.e., VFR. Moreover, the SV/VET ratio was also calculated to obtain VER, which is directly related to myocardial performance^33,34^.

Echocardiography images were taken at rest and during the last (i.e., the fifth, when a steady state in HR was achieved, as indicated by a HR level not different from ± 5 bpm with respect to the previous minute of exercise) minute of exercise by the same operator. When images were considered of good quality, a 6 s frame was recorded and then analyzed offline. For each analysis, at least three beats were taken into consideration (range 3–6 beats) and data are reported as the average of the measures. All echocardiographic calculations were performed by the same expert physician, with 5-year experience in the field. The operator’s coefficient of variation of measurements ranged from 8% (very good) to 12% (good), calculated with an online tool (http://www.birmingham.ac.uk/echo) with measures obtained both at rest and during exercise.

A manual sphygmomanometer (Heine Gamma GP, Gilching, Germany) was placed on the non-dominant arm to assess systolic (SBP) and diastolic (DBP) blood pressure. Blood pressure measurements were performed by the same physician throughout all experiments, with three measurements taken at rest and one measure taken during the last minute of exercise. Care was taken that the subject did not grip the bike during measurement. MAP was calculated using a formula which takes into consideration changes in PEP, VET, and DT^57^. SVR was calculated as MAP/CO. This quantity was then multiplied by 80, where 80 is a conversion factor to change the units to standard resistance units.

### Data analysis

Data are presented as mean ± SD. The Kolmogorov–Smirnov test was utilized to verify the normality of the variables. Since all variables were normally distributed, parametric statistical analysis was employed. Differences between groups in their anthropometric characteristics, and in parameters gathered during the CPT test, were determined using the t test for unpaired data. The differences between groups in variables gathered at rest, and at the fifth minute of exercise at the GET intensity, were tested with the two-way analysis of variance (ANOVA, factors: group and time), followed by a Bonferroni post-hoc when appropriate. Furthermore, the % change with respect to rest for each variable during the GET test was calculated, and a comparison between groups was conducted using the t test for unpaired data.

Statistical analysis was carried out with commercially available software (GraphPad Prism). A p value < 0.05 was considered to determine statistical significance in all cases.

### Ethical approval

All procedures performed in studies involving human participants were in accordance with the ethical standards of the institutional and/or national research committee and with the 1964 Helsinki declaration and its later amendments or comparable ethical standards.

## Data Availability

The data that support the findings of this study are available from the corresponding author upon request.

## Abbreviations

Am: Peak velocity of mitral valve motion during late diastole
Avel: Atrial transmitral filling peak velocity
CO: Cardiac output
CPT: Cardiopulmonary exercise stress test
DBP: Diastolic blood pressure
DT: Diastolic time
E/A: Ratio between early and atrial transmitral filling peak velocities
EF: Ejection fraction
EG: Elderly group
EDV: End diastolic volume
Em: Peak velocity of mitral valve motion during early diastole
ESV: End systolic volume
Evel: Early transmitral filling peak velocity
Evel/Em: Ratio between early transmitral filling peak velocity and peak velocity of mitral valve motion during early diastole
GET: Gas exchange threshold
HR: Heart rate
MAP: Mean blood pressure
PEP: Pre-ejection period
PWD: Pulsed-wave Doppler
RER: Respiratory exchange ratio
SBP: Systolic blood pressure
SD: Standard deviation
Sm: Systolic myocardial velocity
SV: Stroke volume
SVR: Systemic vascular resistance
V_E_: Pulmonary ventilation
VER: Mean ventricular ejection rate
VET: Left ventricular ejection time
VFR: Ventricular filling rate
${\dot{\text{V}}}$CO_2_: Carbon dioxide production
$${\dot{\text{V}}}$$O_2_: Oxygen uptake
${\dot{\text{V}}}$O_2max_: Maximum oxygen uptake
W_max_: Maximum workload
YG: Young group
